# Differences in the Treatment of Acute Coronary Syndrome in the Pre-COVID and COVID Era: An Analysis from Two German High-Volume Centers

**DOI:** 10.3390/jcdd8110145

**Published:** 2021-10-30

**Authors:** Dennis Eckner, Eva M. Hofmann, Fadil Ademaj, Kristinko Martinovic, Ferdinand Vogt, Peter Moritz Becher, Benedikt Schrage, Dirk Westermann, Matthias Pauschinger

**Affiliations:** 1 Department of Cardiology, Paracelsus Medical University, 90471 Nuremberg, Germany; EvaMaria.Hofmann@klinikum-nuernberg.de (E.M.H.); Fadil.Ademaj@klinikum-nuernberg.de (F.A.); Kristinko.Martinovic@klinikum-nuernberg.de (K.M.); Matthias.Pauschinger@klinikum-nuernberg.de (M.P.); 2 Department of Cardiac Surgery, Paracelsus Medical University, 90419 Nuremberg, Germany; ferdinand@vogt.net; 3 Department of Cardiac Surgery, Artemed Clinic Munich South, 81379 Munich, Germany; 4 Department of Cardiology, University Heart and Vascular Centre, 20251 Hamburg, Germany; m.becher@uke.de (P.M.B.); b.schrage@uke.de (B.S.); d.westermann@uke.de (D.W.)

**Keywords:** COVID-19 pandemic, acute coronary syndrome, pain to first medical contact, age distribution, subacute myocardial infarction

## Abstract

The COVID-19 pandemic is placing a heavy burden on healthcare systems worldwide with the risk that acute cardiovascular diseases are treated too late. The present study aims to analyze patients with acute coronary syndrome in the current pandemic. A total of 966 patients (2019 *n* = 463, 2020 *n* = 503) can be evaluated. A comparison of patient care during and before the COVID-19 pandemic was made in terms of patient characteristics and pre- and in-hospital processes. Another aim is to show how many patients seek clinical care at a late stage of the disease. After Lockdown in Germany at week 12, 2020, there was a significant decrease in patients with an acute coronary syndrome (ACS), significant for STEMI cases in the first weeks after Lockdown (calendar week 13–16 2019 *n* = 43, 2020 *n* = 30; *p* = 0.02). The time from pain to first medical contact (time to FMC) is significantly extended during Lockdown, while internal clinical processes are unchanged. The rate of subacute myocardial infarction is numerically, but not significantly increased in calendar weeks 15, 2020 (*p* = 0.40) and 16 (*p* = 0,19). In addition, elderly patients avoid treatment for multifactorial reasons, and the longer overall pain to FMC may impact long-term mortality.

## 1. Introduction

After the SARS COVID-19 pandemic outbreak, which was initially described in Wuhan, Hubei Province, China, it has been spread worldwide [1]. Rising case numbers and higher mortality than previous endemic viruses led to widespread reductions in public life in and outside Europe and ultimately to lockdown scenarios. In everyday life in Germany, clear hygiene rules and the renunciation of unnecessary contacts have been proclaimed [2]. Leaving the own apartment is only possible for a valid reason during Lockdown. In Germany, this Lockdown was declared from the end of calendar week 12.

Elective treatments and operations have been postponed in preparation for many COVID 19 patients burdening the clinics. These instructions met with a great deal of multimedia interest and were distributed accordingly. As a result, many centers around the world reported falling patient admissions for cardiovascular diseases and warned of corresponding acute and long-term consequences [3,4,5]. In addition, suggestions were made for the invasive management of acute coronary syndrome in the COVID-19 pandemic [6]. It is very important to ensure proper standard care of health and treatment for all patients in any case.

The data situation concerning to the patient collective, which shows particular reluctance to treat in Lockdown, is rare. Likewise, little has been published about the delayed clinic presentation’ ‘s specific circumstances [3,7].

Therefore, the aim of the present study is to evaluate prehospital and in-hospital processes and to detect patient groups that are particularly affected by the pandemic. This may improve the care of acute coronary syndrome in the current pandemic or future public health challenges.

## 2. Materials and Methods

### 2.1. Study Design

This retrospective analysis shows data of patients who presented between calendar week 1–18 2019 and 1–18 2020 (1.01.–31.04. in both years) with the clinical suspicion of a ST-segment elevation myocardial infarction (STEMI), or non-ST-segment elevation myocardial infarction (NSTEMI) in the cardiology departments of the University Medical Center Hamburg-Eppendorf and the Paracelsus Medical University Klinikum Nuremberg. Invasive coronary diagnostics were performed on all patients in this study. In addition, the recording of patient-, procedure- and intra-clinical parameters took place.

By discriminating the absolute number of cases per calendar week, a decrease can be documented after the proclamation of Lockdown in calendar week 12. Furthermore, attention is drawn to the age and gender distribution during Lockdown compared to the mentioned time period of 2019. Crucial procedure times are also highlighted. The time-to-first medical contact (time-to-FMC) and the in-hospital door-to-balloon time are compared. Concerning possible delayed presentations in the case of an acute coronary syndrome, patients are examined who already had a maximum individual concentration of cardiac markers (high-sensitive Troponin, hsTnT) upon admission to the clinic, defined as an initial high sensitive troponin T value > 0.050 µg/L and an increase of at most ≥0.01 µg/L in the course. The study complies with the Declaration of Helsinki and is approved by our clinic ethics committee (internal registration number SZ_W_162.20-II-8). 

### 2.2. Statistical Analysis

For numerical data, all baseline characteristics and procedural data variables are defined in mean values with min/max and standard deviation (SD). Categorical data are given in n and %. STEMI and NSTEMI cases over the calendar weeks are subdivided into weeks 1–18, 12–18, and 13–16; the differences are given in % and the respective *p*-value.

The age distribution is analyzed in decades and is normalized to 10 cases per week. The evaluation is also carried here with the corresponding *p*-value. The process differences of pain-to-FMC and door-to-balloon time are shown in the median/week due to better visual representation with a reduction of outliers. The static evaluation is carried out after checking for normal distribution (Kolmogorov Smirnov test); for continuous variables, we use the t-test. Categorical variables are analyzed using the Mann-Whitney test and binary data using the chi-square test. If more than 5% of the data is missing, missing data will be ignored in the analyses and reported in the results.

The data processing and visualization was performed with XL-Stat (Addinsoft, New York, NY, USA), Microsoft Excel (Redmond, WA, USA), and SPSS version 24 (Chicago, IL, USA).

## 3. Results

We could identify 463 patients with acute coronary syndrome in weeks 1–18 of 2019 (mean age 67.6 years, 27.4% women). During the same period of 2020, there were 503 patients (+8.6%, mean age 67.6 years, 28.2% women).

After Lockdown in Germany at the end of the 12th calendar week of 2020, 8.28% fewer patients with STEMI and NSTEMI presented compared to 2019 (145 patients in 2019, 133 patients in 2020). Differentiated by entity in this period, it can be stated that the number of NSTEMI patients ‘didn’t differ significantly (2019 *n* = 86, 2020 *n* = 90, +4.65%, *p* = 0.86), while the rate of STEMI cases decreased by 27.12% (2019 *n* = 59, 2020 *n* = 43, *p* = 0.06). Especially in the first three weeks after Lockdown, an even more evident decrease in both entities can be seen from week 13–16 (*p* for NSTEMI 0.09, *p* for STEMI 0.02; 2019 *n* = 43, 2020 *n* = 30).

Concerning previous and concomitant illnesses in 2020, the patients have significantly more diabetes mellitus and more often hypercholesterolemia (DM 26.9% vs. 33.6%, *p* = 0.02, hypercholesterolemia 53.1% vs. 59.4%, *p* = 0.05). Furthermore, no relevant differences in the patient groups can be documented. In particular, the LV function is not significantly different (2019 48.2% vs. 47.7%, *p* = 0.58; Table 1).

A STEMI will be treated in 34.8% in 2019 and in 33.2% in 2020 (*p* = 0.64). The remaining cases are in NSTEMI patients (65.2% vs. 66.8%, *p* = 0.64).

The procedure time in the cardiac catheterization laboratory, the initial hemodynamic parameters and the initial and maximum troponin T and CK values are not significantly different (Table 2).

An initial cardiogenic shock is present in 4.9% in 2019, and in 5.2%, the use of an MCS is necessary. In 2020, the rate of cardiogenic shock is 6.2%; an MCS is implanted in 5.4% (*p* for CS 0.48, for MCS 1.00). 

Intra-hospital mortality is not significantly different over the entire period (2019 6.3% vs. 6.8% in 2020, *p* = 0.79).

Overall, a significantly shorter hospital stay in 2020 can be seen, while the duration of the ICU stay ‘doesn’t differ significantly (2019 8.2 days and 2.7 days on ICU, 2020 6.9 and 2.2 days, *p* < 0.01 and 0.08).

The analysis of the number of cases over the calendar weeks 2020 shows a significant decrease after the Lockdown in calendar week 12.

**Figure 1:**
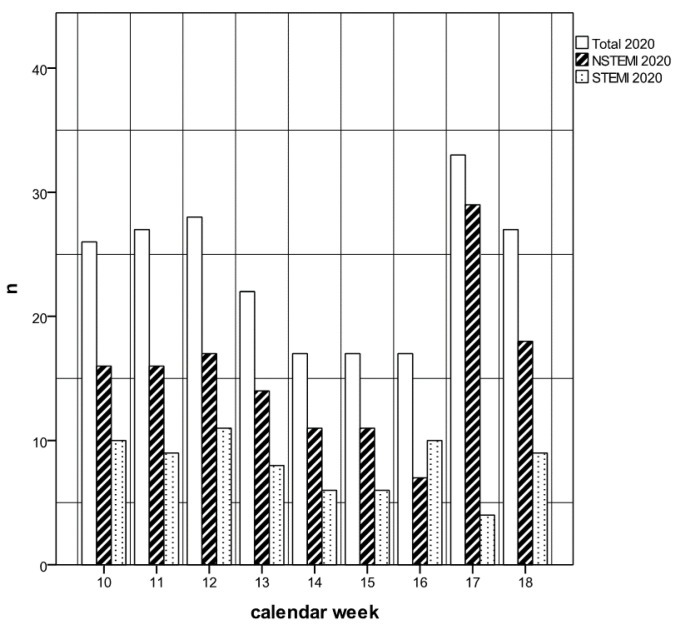
Cases over calendar weeks 2020. After the start of the Lockdown at week 12, STEMI and NSTEMI cases decreased. Resumption from week 17.

This decrease in the number of cases compared to 2019 can be seen numerically in both NSTEMI and STEMI patients, but this is significant in STEMI (overall cases *p* < 0.01, STEMI *p* = 0.02, NSTEMI *p* = 0.09). From calendar week 17, the number of NSTEMI cases increases again and reaches a level before the Lockdown (Figure 1).

**Figure 2:**
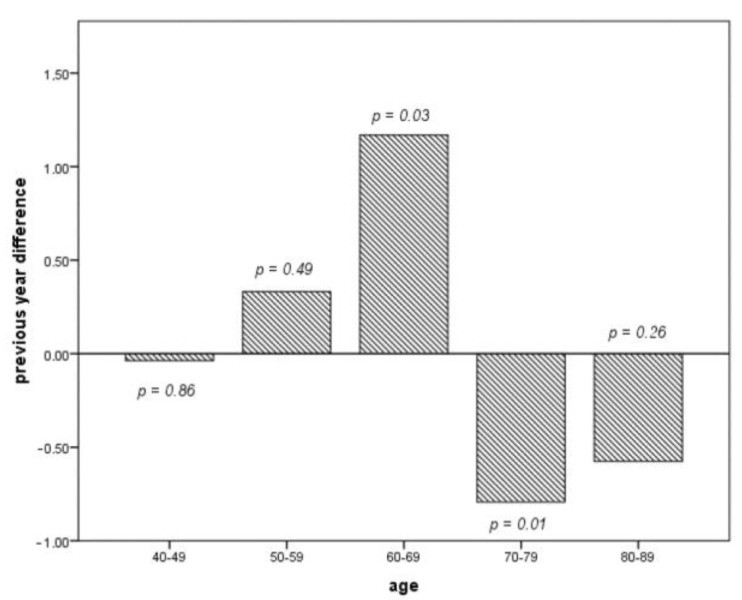
Comparison of the case difference by age from week 12–18 in 2019 and 2020 (each per 10 cases). Fewer cases > 70 years in favor of younger patients.

Focusing on the age of the patients who present with an ACS in Lockdown, it can be seen that, compared to 2019, those between 60 and 69 years of age present themselves significantly more often (*p* = 0.03) and between 70–79 (*p* = 0.01) years substantially less frequently. This trend is also evident in patients over 80 years of age (Figure 2).

In contrast, there was no difference between 2019 and 2020 in terms of gender differences at clinical presentation; no significant difference can be seen over the entire period or in the lockdown weeks (*p* for calendar week 1–18 =0.71, *p* for calendar week 12–18 =0.80, *p* for calendar week 13–16 =0.39).

The preclinical and intra-clinical process duration in STEMI patients shows significant differences in the pandemic.

We examine two process metrics in STEMI patients. On the one hand, the time from the first cardiac pain event to first medical contact (pain-to-FMC), on the other hand, the time from clinical admission to balloon angioplasty of the coronary culprit lesion (door-to-balloon time) is examined.

For the pain-to-FMC, 72% of the data records that can be fully evaluated are available in 2019 and 79% in 2020. The door-to-balloon time is known in 2019 at 59.6%, in 2020 at 67.7%.

**Figure 3:**
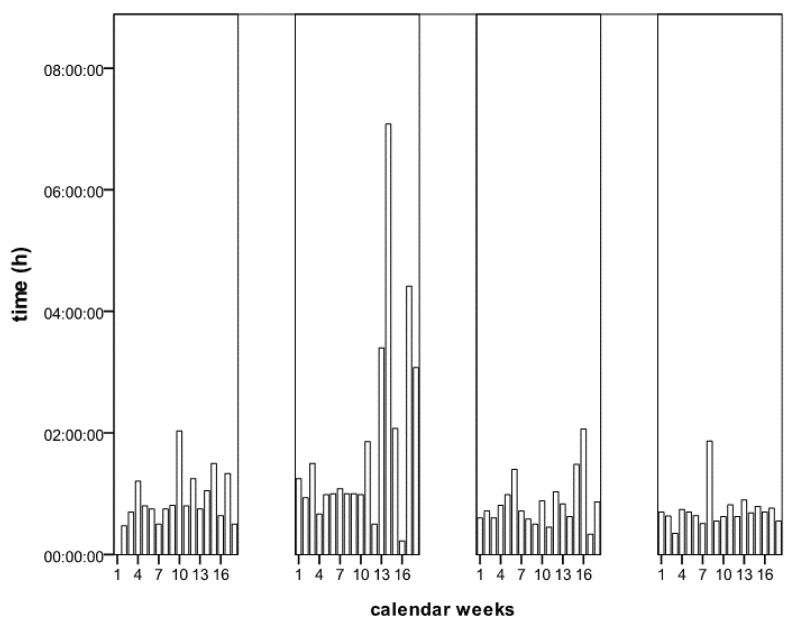
Comparison of the “pain-to-first medical contact” and “door-to ballon” time 2019 and 2020 (each data are given as median).

First of all, concerning the pain-to-FMC, it can be shown that this is the same over the entire observation period (54.5 vs. 63 min, *p* = 0.19). After lockdown start, the process was prolonged numerically from week 12–18 (60 vs. 100 min, *p* = 0.10), and from week 13–16, a significant process extension was observed (57.5 vs. 108 min, *p* = 0.02, Figure 3).

The in-hospital processes in STEMI patients show no delay in the pandemic. Instead, it can be seen that with a reduced number of patients, treatment is numerically faster after week 12 (week 12–18 60 vs. 42 min, Figure 3).

**Figure 4:**
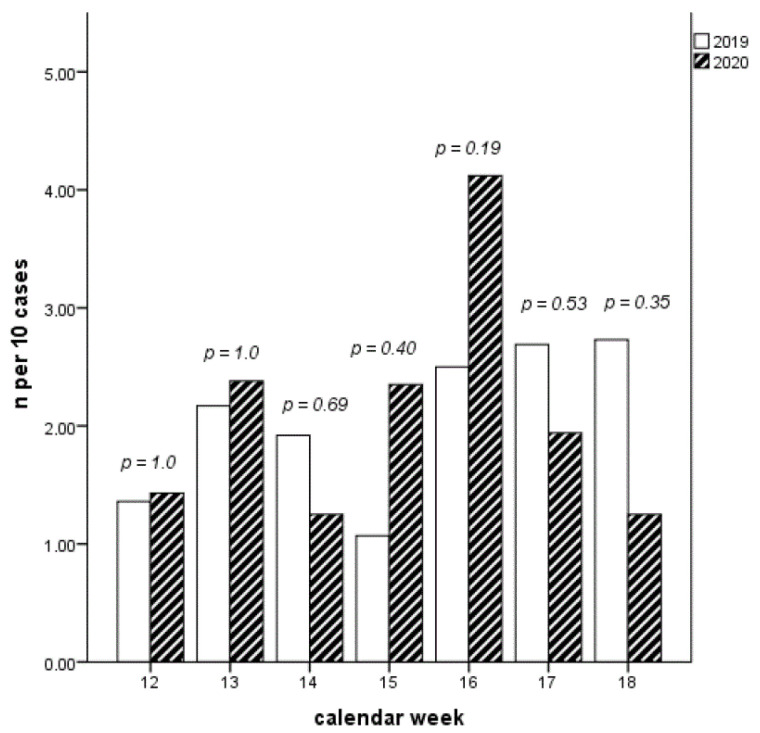
Number of cases with maximum high sensitive TroponinT already achieved at admission over calendar weeks 12–18. Numerically increased presentation of patients with already maximal Troponin in LLockdown as an expression of a delayed presentation.

Finally, patients who presented at a later stage of disease were examined. This delay is defined as the maximum hsTnT concentration already achieved upon admission to the hospital. In week 12–16 of 2020 there was a numerical increase of patients with a maximum hsTnT. This trend is reversed analogously to the higher total numbers from week 17 onwards. A statistical significance of this phenomenon cannot be shown over the weeks (Figure 4).

## 4. Discussion

The COVID-19 pandemic has dominated the medical world since the beginning of 2020. In Germany, this poses a particular challenge to hospitals in the face of ubiquitous staff shortages. Studies worldwide describe the decline in cardiovascular and emergency patients in other disciplines [6,8,9,10,11,12].

Concerning myocardial infarction, this decline in patients is different depending on the pandemic intensity of the countries and regions.

While regions severely affected by the pandemic, such as northern Italy, have seen a massive drop in cardiac patient numbers, the decline in the number of cases in other analyses and ours is more moderate in terms of the total number.

Our analysis shows a decrease in patient presentations after the Lockdown in Germany at the end of the calendar week 12. The rate of NSTEMI cases recovers from calendar week 17 and reaches a level overall as in 2019, while fatally, the STEMI presentations decrease by a total of 27.12% and at most stabilize again from calendar week 18 on.

There is not only a threatening reduction in the total number of STEMI patients. STEMI also shows a delay in presentation, defined as pain-to-FMC, which in the long term can contribute to increased morbidity and mortality in the treated cases with more significant myocardial damage [13,14].

Since most clinics in Germany managed to function without overloading the clinical structures in the first wave of the pandemic through appropriate preparations, immediate care of STEMI patients is guaranteed within the clinic despite massive additional workload from isolated COVID-19 patients. This is also shown by our investigation with an even accelerated door-to-balloon time in Lockdown with a lower overall number of cases. However, it should be noted, that this only appears to be possible up to a critical number of COVID-19 patients and otherwise this quality of care can also experience restrictions.

About the late presentation of ACS patients and a look at the cardiac enzymes in our data, a non-significant but numerically increased number of patients visit the clinic at a very late point of the ACS can be seen. In the context of long-term consequences with the possible development of ischemic heart disease, an impact on the long-term survival of many patients may be possible [13,15].

Therefore, it is crucial to maintain these multifactorial process structures, which are ensured preclinically by the resident colleagues and the professional rescue service and are continued in the clinic and provide sufficiently efficient patient care. Despite intensive care medicine focusing on COVID-19 patients, sufficient capacities should be available to continue to care for critically ill ACS patients.

Our data also shows that especially patients over 70 years of age have more restrictive behavior concerning a clinic presentation. Fatally, studies have shown an exceptionally high ACS mortality in this age group [16,17]. This phenomenon must be discussed; whether the multimedia attention to risk groups for a severe COVID-19 course in this age and the urgent political call for “stay at home” has led to an increased reluctance of the patients with regard to a clinical presentation. Meanwhile, existing data indicate that at the beginning of the pandemic, a relevant proportion of infections still took place in the hospital, while a low nosocomial infection rate is currently recorded [18,19]. In Germany, however, most of the sources of infection are still unknown.

However, we were unable to show the effect of gender on the clinical presentations.

Overall, it should be discussed whether more intensive cardiological education and political public relations work can help reverse this trend to maintain rapid, guideline-based therapy in this sensitive collective. In the future, it will also be necessary to modify and establish other systems to ensure quick and efficient access to the population. First of all, it is essential to ensure sufficient staffing of the state health service. In addition, regional and supraregional government agencies can help to provide targeted information to population groups utilizing web-based assistance, for example. This is already being carried out in the current pandemic through Corona Warn apps. Therefore, it would be desirable to use similar systems for the prevention of cardiovascular events.

The limitations of the present study about the differences in mortality may be due to the small number of patients who died in the hospital. The same applies to the number of cardiogenic shock patients observed.

It should also be noted that the available data is only based on the patients who were subject to a cardiac catheter examination. Therefore, this does not include all those patients who presented clinically due to other cardiac events such as heart failure or arrhythmias.

However, it is known from other studies that the number of patient presentations for other cardiovascular reasons has also decreased in the pandemic [20,21].

This decrease is because elective interventions had to be postponed to a later date because of political requirements.

The effects of these postponed interventions on the forecast must be clarified in the future.

Furthermore, future studies must precisely analyze ‘patient’s long-term prognosis develops who were treated with the deficits described in Lockdown in ACS.

## 5. Conclusions

The COVID-19 pandemic places particular demands on the care of patients with acute coronary syndrome. A decreasing number of patients in Lockdown can be observed mainly in older patients and, unfortunately, in the STEMI collective, which makes the need for intensive medical and political education necessary to ensure adequate acute care. Furthermore, under all circumstances, especially in the current situation, it must be ensured that preclinical and intra-clinical processes continue to function optimally to minimize a further delay until the important revascularization of the infarct vessel and to provide our patients with current guideline-based therapy.

## Figures and Tables

**Table 1:** Baseline characteristics.

	2019 (*n* = 463)			2020 (*n* = 503)			*p*
	Mean	min/max/SD	%	Mean	min/max/SD	%	
Age (years)	67.6	17–95, 5.3		67.6	19–95, 12.2		0.97
Sex female, no. (%)			27.4			28.2	0.83
BMI	27.7	13.8–67, 5.3		27.6	18.6–53, 4.5		0.66
Arterial hypertension			74.3			77.9	0.21
DM			26.8			33.6	0.02
Hypercholesterolemia			53.1			59.4	0.05
Renal failure			34.8			35.4	0.89
Pulmonary hypertension			5.6			7.8	0.19
Atrial fibrillation			14.7			16.3	0.53
PAD			14.5			16.3	0.48
Current smoker			27.7			26.0	0.62
CABG			6.9			9.5	0.16
Pre-PCI			17.1			20.1	0.24
Apoplexy			7.6			8.5	0.63
Pre-MI			13.6			15.7	0.37
LV-EF	48.2	10–76, 13.8		47.7	5–73, 13.2		0.58

Data are expressed as mean ± standard deviation or percent (%). (BMI = body mass index, DM = diabetes mellitus, PAD = peripheral arterial disease, CABG = coronary artery bypass graft, pre-PCI = previous percutaneous coronary intervention, pre-MI = previous myocardial infarction, LV-EF = left ventricular ejection fraction).

**Table 2:** Peri- and postprocedural characteristics and outcome.

	2019 (*n* = 463)		2020 (*n* = 503)		*p*
	*n*	% or Mean	*n*	% or Mean	
STEMI	161	34.8	167	33.2	0.64
NSTEMI	302	65.2	336	66.8	0.64
Cardiogenic Schock	23	4.9	31	6.2	0.48
MCS (IABP, Impella, ECMO)	24	5.2	27	5.4	1.00
Radial approach	290	62.6	335	66.6	0.21
Procedure time (min)		56.3		55.3	0.67
Heart rate (per min)		78.0		79.5	0.19
BP mean (mmHg)		90.5		90.1	0.80
Initial hsTnT (ng/mL)		0.6		0.7	0.62
Initial CK (U/L)		388.3		438.1	0.41
Max hsTnT (ng/mL)		2.1		2.1	0.82
Max CK (U/L)		1274.9		1276.3	0.99
Peripheral vascular complication	18	3.9	14	2.8	0.38
Death in cath lab	2	0.4	3	0.6	1.00
Intrahospital death	29	6.3	34	6.8	0.79
Mortality of CS	13	56.5	22	71	0.39
ICU stay (d)		2.7		2.2	0.08
Hospital stay (d)		8.2		6.9	<0.01

Data are expressed as mean or total number with percent (%) including the *p* value for each variable. (CS = cardiogenic schock, MCS = mechanical assist system, IABP = intra-aortic ballon counterpulsation, ECMO = extra-corporeal membrane oxygenation, BP = blood pressure hsTnT = high-sensivite TroponinT, CK = Creatinkinase, ICU = intensive care unit, d = days).

## Data Availability

The data presented in this study are available on request from the first author. The data are not publicly available due to data protection regulations.
